# Computed Tomography Assessment of Brain Atrophy in Centenarians

**DOI:** 10.3390/ijerph16193659

**Published:** 2019-09-29

**Authors:** Robert Chrzan, Agnieszka Gleń, Amira Bryll, Andrzej Urbanik

**Affiliations:** Department of Radiology, Jagiellonian University Medical College, Kopernika 19, 31-501 Krakow, Poland; agaglen@wp.pl (A.G.); bryllamira@gmail.com (A.B.); andrzej.urbanik@uj.edu.pl (A.U.)

**Keywords:** centenarians, brain atrophy, computed tomography

## Abstract

The aim of our study was to compare the degree of brain atrophy in centenarians and in seniors 70–99 years old. The study group consisted of 23 patients aged 100–106 years. The control group consisted of 90 patients, 30 in each age subgroup 90–99, 80–89, 70–79. In all the patients, the brain atrophy linear parameters were measured on computed tomography scans, in relation to both “subcortical atrophy”, evaluated as progressive widening of the ventricular system, and “cortical atrophy”, defined as widening of subarachnoid space. Secondary indices based on the parameters were calculated. Correlations between the above parameters/indices and age were tested. Significantly different values between the centenarians and the control group were found in the brain atrophy parameters: A, B, C, E, FI, ICR, ICL, SW, CFW, F/A ‘frontal horn index’, A/G ‘Evans index’, D/A ‘ventricular index’, H/E ‘cella media Schiersmann index’, A+B ‘Huckman number’. Correlations between parameter/index and age were found for: A, B, C, FI, ICR, ICL, SW, F/A ‘frontal horn index’, A/G ‘Evans index’, D/A ‘ventricular index’, H/E ‘cella media Schiersmann index’, A+B ‘Huckman number’. Brain atrophy associated with aging is a continuously advancing process, affecting centenarians even more than people before the “magic” threshold of 100 years.

## 1. Introduction

It is expected that the older adult population of the world will grow significantly in the following decades. According to the Population Division of the United Nations (UNDP) [1], compared to 2017, the number of people aged 60 or over is expected to more than double by 2050 and more than triple by 2100, rising from 962 million in 2017 to 2.1 billion in 2050 and 3.1 billion in 2100. Moreover, the number of people aged 80 or over is projected to triple by 2050, and subsequently by 2100 to increase to nearly seven times its value in 2017, rising from 137 million in 2017 to 425 million in 2050, and further to 909 million in 2100.

The UNDP estimated the global number of centenarians (people aged 100+) to be 451,000 in 2015 and forecasted that number to be multiplied 50 times in 2100 reaching more than 25 million [2]. Such a rapid increase in the number of older people significantly contributes to the growing interest in research of the aging human brain, including both “physiological” changes and the diseases common at advanced age, resulting in dementia, such as cerebrovascular disease (CVD) or Alzheimer’s disease (AD).

Progressive changes in the structure and function of the brain [3], particularly involving cognitive impairment [4], are typical for aging. However, in some older people, this process is significantly slowed down. That is why the terms “normal brain aging” versus “healthy brain aging” have been introduced [5].

Normal brain aging describes the age-dependent changes in the structure and function of the brain but with no clinically important impairments, observed in the general population. To the contrary, healthy brain aging is defined as preservation of the structure and function of the brain in spite of aging and is found far less frequently. Centenarians may be regarded as a “lucky” subpopulation with the aging process significantly slowed down, because of genetically inherited and lifestyle related factors. Thus, the question arises whether brain atrophy in centenarians is also substantially slowed down, like in healthy brain aging, or perhaps it is a continuously advancing process that affects centenarians even more than people before the “magic” threshold of 100 years.

The aim of our study was to compare the degree of brain atrophy in centenarians and in seniors aged 70–99 years.

## 2. Materials and Methods

The study protocol had been reviewed and approved by the local Bioethics Committee.

The hospital picture archiving and communication system (PACS) was searched for brain CT (computed tomography) examinations performed in patients aged at least 100 years, during the period from year 2012 to 2017.

In each case, the age was confirmed by checking the patient’s identification number PESEL (Universal Electronic System for Registration of the Population) to exclude erroneous values resulting from incorrect registration or unknown personal data at admission.

Only cases with no hematoma, brain tumor, acute stroke, or post-infarction encephalomalacia resulting in mass effect or asymmetry, affecting the ventricular system shape or subarachnoid space volume, were selected for the study group.

The search finally identified a group of 23 patients—15 females and eight males, aged 100–106 years (mean 102 years, SD (standard deviation) 1.8 years).

Unfortunately, no psychological tests assessing cognitive impairments were available in the patients’ electronic medical records.

Three control groups were then created, using PACS to search for brain CT examinations performed in 2017, and by identifying the first 30 consecutive patients representing the following age ranges: 90–99, 80–89, and 70–79, without suspected dementia listed on the CT referral form, suggesting severe brain atrophy, and with no hematoma, brain tumor, acute stroke, or post-infarction encephalomalacia resulting in mass effect or asymmetry, affecting the ventricular system shape or subarachnoid space volume.

The group aged 90–99 consisted of 22 females and eight males (mean 93 years, SD 2.2 years).

The group aged 80–89 consisted of 18 females and 12 males (mean 84 years, SD 2.8 years).

The group aged 70–79 consisted of 15 females and 15 males (mean 74 years, SD 2.6 years).

All the CT examinations were performed using multirow (16, 64, or 80 rows) helical scanners, slice thickness 2, 2.5 mm or 3 mm slices, 120–130 kV, 102–342 mAs.

**Figure 1:**
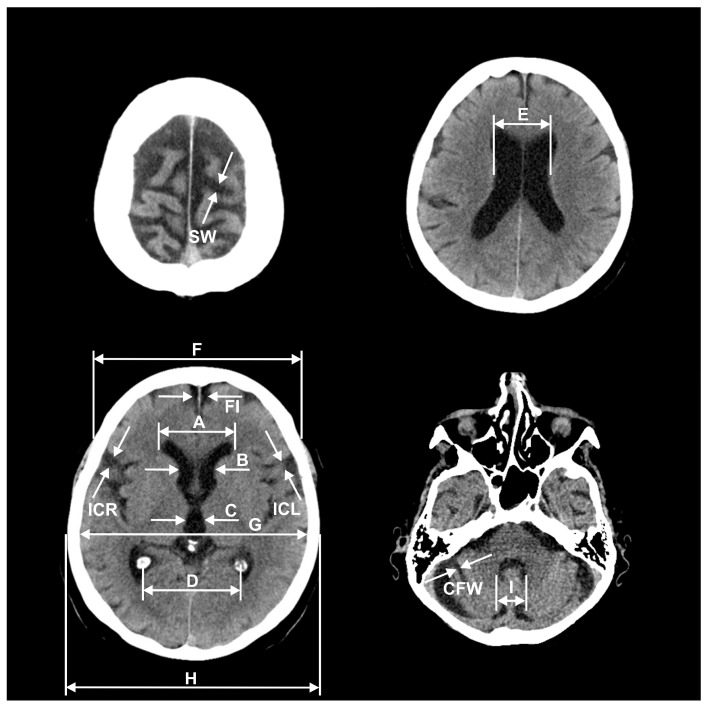
Measurements of brain atrophy parameters: A, B, C, D, E, F, G, H, I, FI, ICR, ICL, SW, CFW in CT images.

In all the patients, the brain atrophy parameters were measured on the CT scans, based on the commonly-used method described by Meese [6] (Figure 1).

Every parameter was measured twice and the mean value was calculated to increase accuracy and limit the “partial volume” effect.

The parameters: A, B, C, D, E, and I estimated the intensity of subcortical atrophy by the ventricular system measurements (in mm) and, respectively, referred to: the frontal horns greatest distance, the caudate nuclei distance, the third ventricle width, the choroid plexuses distance, the lateral ventricles greatest distance at the level of cella media, and the fourth ventricle width.

The parameters FI, IC, SW, and CFW estimated the intensity of cortical atrophy by the subarachnoid space measurements (in mm) and, respectively, referred to: the longitudinal cerebral fissure width in anterior part, the insular cisterns width (ICR, ICL—separately for the right and left side), the cerebral sulci greatest width at the skull vault, and the cerebellar fissures’ greatest width.

The parameters: F, G, and H were used for the calculation of indices by the cranial vault bones measurements (in mm) and, respectively, referred to: the frontal bones’ greatest external distance, the temporal bones’ greatest internal distance, and the temporal bones’ greatest external distance.

The calculated indices included F/A, A/G, D/A, H/E, and A + B and, respectively, referred to: ‘the frontal horn index’, ‘the Evans index’, ‘the ventricular index’, ‘the cella media Schiersmann index’, and ‘the Huckman number’.

Mean values of the parameters and indices were obtained independently for the group of patients aged 100–106, for the control groups of patients aged 90–99, 80–89, and 70–79, respectively, as well as for the entire control groups—all the patients aged 70–99.

Statistical significance of differences between the mean values was evaluated using: Student’s t-test for independent samples, Welch’s t-test (if variances equality was not confirmed using Levene’s test), and Mann-Whitney’s test (if the distribution of a feature was not normal).

The Shapiro-Wilk’s test was used to assess agreement between the distribution of a feature and a theoretical normal distribution.

Additionally, the above parameters/indices were tested for correlations with age, in the group of all the patients aged 70–106, using Pearson’s r correlation coefficient, with correlation linear formula automatically calculated by the least squares method.

The statistical calculation was performed using Statistica software version 13.3 (TIBCO Software Inc.).

The study has been approved by the bioethics committee of the Jagiellonian University in Kraków (opinion No. 1072.6120.316.2018 of December 20, 2018). The study was conducted in accordance with the ethical principles of the Helsinki Declaration of 2008. The bioethics committee decided that there was no need to use a written study participant’s consent form, because of retrospective assessment of CT images.

## 3. Results

Mean values of brain atrophy parameters and indices in the consecutive age groups are presented in Table 1.

The statistically significant (*p* < 0.05) differences were as follows:

In the group of 100–106 years old subjects the mean values of A (43.00 mm), A/G Evans index (0.32) and A + B Huckman number (68.74 mm) were significantly higher from the corresponding values in all the control groups: 90–99 years old (38.77 mm, 0.29, 63.57 mm, respectively), 80–89 years old (38.17 mm, 0.29, 60.60 mm), 70–79 years old (37.20 mm, 0.27, 57.90 mm), as well as 70–99 years old (38.04 mm, 0.28, 60.69 mm).

In the group of 100–106 years old subjects the mean values of F/A frontal horn index (2.94) and D/A ventricular index (1.36) were significantly lower from the corresponding values in all the control groups: 90–99 years old (3.27, 1.53, respectively), 80–89 years old (3.33, 1.57), 70–79 years old (3.47, 1.60), as well as 70–99 years old (3.36, 1.56).

In the group of 100–106 years old subjects the mean values of B (25.74 mm), FI (8.87 mm), ICR (11.35 mm), ICL (11.83 mm), SW (7.30 mm), and CFW (3.83 mm) were significantly higher from the corresponding values in the control groups: 80–89 years old (22.43 mm, 7.20 mm, 8.03 mm, 8.70 mm, 5.60 mm, 2.87 mm, respectively), 70–79 years old (20.70 mm, 6.00 mm, 7.17 mm, 7.33 mm, 5.47 mm, 2.60 mm), as well as 70–99 years old (22.64 mm, 7.23 mm, 8.50 mm, 8.86 mm, 5.88 mm, 2.94 mm).

In the group of 100–106 years old subjects the mean values of C (11.48 mm) and E (34.91 mm) were significantly higher from the corresponding values only in the control group of 70-79 year olds (8.70 mm, 29.53 mm, respectively).

In the group of 100–106 years old subjects the mean value of H/E cella media Schiersmann index (4.32) was significantly lower from the corresponding value only in the control group of 70–79 year olds (5.32).

Obviously, there were no statistically significant differences between F, G, and H values in the consecutive age groups, as specified above, because these are cranial vault bones measurements, not affected by brain atrophy, and used only for indices calculations.

No statistically significant differences were found between brain atrophy parameters D and I in the consecutive age groups, as above.

**Figure 2:**
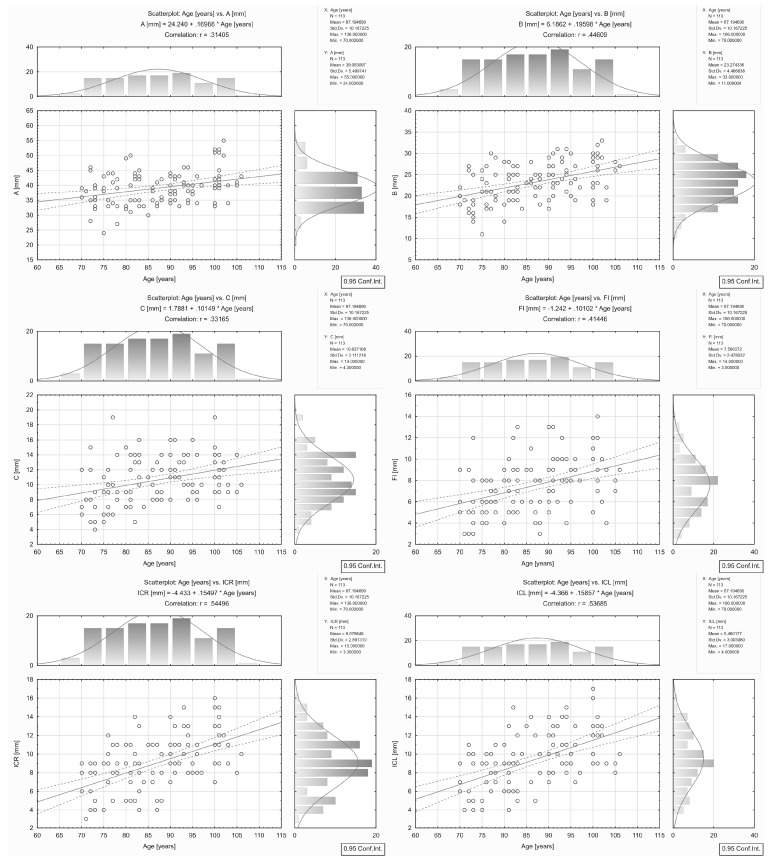
Correlations between brain atrophy parameters/indices: A, B, C, FI, ICR, ICL, and age in the group of all the patients aged 70–106.

**Figure 3:**
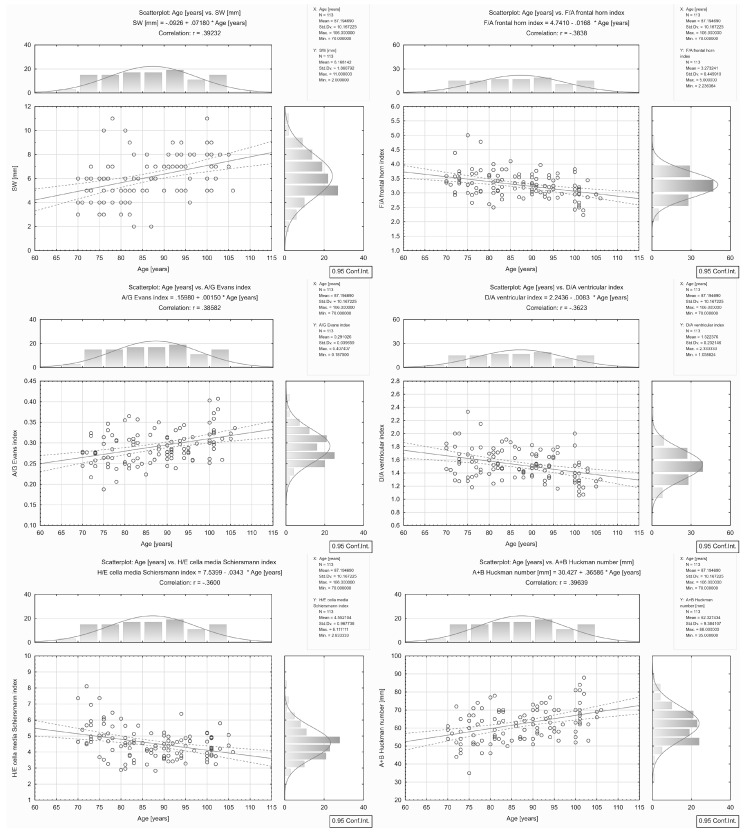
Correlations between brain atrophy parameters/indices: SW, F/A, A/G, D/A, H/E, A + B and age in the group of all the patients aged 70–106.

The correlations found between brain atrophy parameters/indices and age are presented in Table 2 and Figure 2 and Figure 3.

A strong correlation (|*r*| coefficient > 0.5) between parameter/index and age was found for ICR (*r* = 0.54) and ICL (*r* = 0.54).

A moderate correlation (0.5 ≥ |*r*| > 0.3) between parameter/index and age was found for B (*r* = 0.45), FI (*r* = 0.41), A+B Huckman number (*r* = 0.40), SW (*r* = 0.39), A/G Evans index (*r* = 0.39), F/A frontal horn index (*r* = -0.38), D/A ventricular index (*r* = −0.36), H/E cella media Schiersmann index (r = −0.36), C (*r* = 0.33), and A (*r* = 0.31).

For CFW, D, E, and I, the correlation coefficient |*r*| was ≤ 0.3.

## 4. Discussion

The results obtained in the current study confirmed that brain atrophy in centenarians is more severe compared to people aged 70–99. This applies to both “subcortical atrophy” evaluated as progressive widening of the supratentorial ventricular system and “cortical atrophy” defined as widening of subarachnoid space.

Goldstein [7], using CT, also found more severe brain atrophy in a group of 10 centenarians aged 100–102 years compared to the control group aged 61 to 77.

In his study, the mean frontal horn index 2.85 (1/0.351) in the group of centenarians was significantly lower from the value 3.37 (1/0.297) identified in the control group. This is consistent with our result of 2.94 in the group of centenarians and 3.47 in the control group aged 70 to 79.

In Goldstein’s study, the mean width of the third ventricle amounting to 9.3 mm in the group of centenarians was significantly greater than the value of 6 mm found in the control group. Similarly, our result of 11.48 in the group of centenarians was significantly greater than the corresponding value of 8.70 in the control group aged 70 to 79.

However, in Goldstein’s study, the mean cella media index of 3.86 (1/0.259) was close to the value of 3.92 (1/0.255) in the control group. In our study, the value of 4.32 in the group of centenarians was lower than the corresponding value of 5.32 in the control group aged 70 to 79, but the difference was not statistically significant.

No statistically significant differences found for D (the choroid plexuses distance) can be explained by the fact that in the course of subcortical atrophy, lateral ventricles widen both in the medial and lateral directions, but the position of plexuses in the central parts remains constant.

The fact that no significant differences were found for I (the fourth ventricle width) may be the effect of much slower rate of cerebellar aging compared to the other parts of brain, which is of clinical/biological importance. Horvath [8], using genetic tests in a group of supercentenarians (subjects who reached an age of 110 or older) and younger subjects, demonstrated that the cerebellum ages more slowly than other parts of the human body. Similarly, Fraser [9] found that gene expression patterns in the human cerebellum show less age-related changes compared to the cerebral cortex.

For decades, CT has been commonly used as the first step brain diagnostic imaging method. It is a widely available and fast technique and it may be performed in patients with contraindications for MRI (magnetic resonance imaging), such as ferromagnetic implants or cardiostimulators. However, MRI is now regarded as a much more valuable method for examining the central nervous system [5]. It does not only enable more accurate morphological assessment concerning white and gray matter differentiation or focal lesion assessment, but it also provides data concerning functional disturbances. Unfortunately, in our study, only CT imaging was available.

DeCarli [3] presented one of the earliest and largest studies concerning age-related brain atrophy, based on MRI exams of the Framingham Heart Study participants, i.e., 2200 individuals from 34 to 97 years of age. In his study, changes related to age were slight in people before the age of 50; however, they intensified with age. The greatest decline with age was found in frontal lobe volumes (0.16% of total cranial volume per year in men, and 0.15% in women); the temporal lobes showed smaller changes, while in the occipital and the parietal lobes, age-related differences were modest.

Research investigating independently measured white and gray matter volumes also confirmed associations with age.

Fotenos [10] performed one of the earliest and largest researches of brain atrophy related to age, based on MRI in a group of 370 adults aged 18–97 years. In his study, reduction of whole-brain, gray matter and white matter volumes in non-demented subjects was found at about 0.45 % per year. The intensity of atrophy differed depending on the region (the greatest in the frontal lobes) and the type of tissue.

Raz [11], using MRI in a group of 127 adults aged 31–83 years of age, discovered significant age-dependent loss of white matter volume concerning prefrontal and inferior parietal regions. He also found acceleration of prefrontal white matter atrophy with age, which implies special vulnerability to age-related changes.

MRI also enables assessment of age-dependent gray matter shrinkage, reflected by measurements of cortical thickness.

Lemaitre [12] performed MRI in 216 adults aged 18–87 and measured gray matter volume, surface area and cortical thickness in various brain regions. He discovered age-dependent reductions in almost all gray matter measures.

Van Velsen [13] carried out a study involving 1022 non-demented elderly subjects (mean age 68.4 ± 7.3 years) and also found cortical thickness decreasing with age (approximately 0.2% per year), with the largest age-related effects observed in the occipital and temporal lobes, and a more visible decrease in the frontal lobe in men than in women.

Taking into consideration the fact that memory impairment is common at advanced age and that hippocampal damage is found early in Alzheimer’s disease [14], it is not surprising that hippocampus measurements are reported in many studies focusing on brain aging. Most authors, using MRI, found the reduction in volume of hippocampus with advancing age, even if few people retain “normal” cognitive functions [15,16,17]. Hippocampal volume loss was found to be severe from 60 years onward, with magnitude greater than that of the total cortical volume [15,18]. However, tau pathology concerning hippocampus and other subcortical structures may precede for many years the amyloid pathology typical for Alzheimer’s disease [19].

Yang [17] analyzed age-associated differences on structural brain MRI in non-demented individuals from 71 to 103 years (70 participants ≥ 90 years and 207 < 90 years old). He found negative linear relationships of age with cortical and subcortical gray matter volume, presenting the greatest age effects in the medial temporal lobe as well as parietal and occipital cortices. Of particular interest was the fact that age had a stronger negative relationship with hippocampal volume alone than with total gray matter volume. Yang also found significant negative quadratic effects of age for total white matter volume and positive quadratic effects for white matter hyperintensities volume.

A study performed by Gong [20] applied MRI datasets and sophisticated software based on spherical harmonics (SPHARM) shape analysis pipeline, and analyzed ventricle shape in centenarians, elderly subjects, as well as MCI (mild cognitive impairment) and AD (Alzheimer’s Disease) patients. He found that in spite of ventricle volume increase resulting from atrophy, the difference in ventricle shape between the centenarian group and normal aging group was minor. However, the difference in shape between the centenarian group and AD group brains was significant.

Certainly, even the most sophisticated imaging techniques cannot fully match neuropathological examinations. In a study performed by Ganz [21], 40 centenarians self-reported to be cognitively healthy at baseline; however, in post-mortem neuropathological tests, the hallmarks related to age and neurodegenerative diseases were abundantly present in most of the subjects.

In addition to using CT rather than MRI, another disadvantage of our study was related to the lack of standard psychological dementia tests performed in the centenarians involved. Therefore, we cannot link in any way the brain atrophy found to possible cognitive impairment in the group.

## 5. Conclusions

The final conclusion resulting from our study is the statement that brain atrophy associated with aging is a continuously advancing process, affecting centenarians even more than people before the “magic” threshold of 100 years. This applies to both “subcortical atrophy” evaluated as progressive widening of the supratentorial ventricular system and “cortical atrophy” defined as widening of subarachnoid space.

No significant differences found for the fourth ventricle width may be the effect of much slower rate of cerebellar aging compared to the other parts of brain, which is of clinical/biological importance.

It would be desirable to perform a study including MRI exams and psychological tests, and involving a larger group of Polish centenarians, as a rational development of our retrospective CT study based on a small number of subjects, given the fact that according to GUS (central statistical office), the number of such people in Poland exceeded 4900 in 2017.

## Figures and Tables

**Table 1:** Mean values of brain atrophy parameters and indices in the consecutive age groups.

Parameters	100–106 Years Old	90–99 Years Old	80–89 Years Old	70–79 Years Old	70–99 Years Old
Age [years]	101.52 (100–106, SD 1.75)	92.63 (90–97, SD 2.24)	83.63 (80–88, SD 2.77)	74.33 (70–78, SD 2.56)	83.53 (70–97, SD 7.92)
A [mm]	43.00 (33–55, SD 6.13)	**38.77** **(33–46, SD 3.80)**	**38.17** **(30–50, SD 5.27)**	**37.20** **(24–46, SD 5.41)**	**38.04** **(24–50, SD 4.87)**
B [mm]	25.74 (18–33, SD 4.17)	24.80 (17–31, SD 3.77)	**22.43** **(14–28, SD 3.60)**	**20.70** **(11–30, SD 4.68)**	**22.64** **(11–31, SD 4.34)**
C [mm]	11.48 (7–19, SD 2.71)	11.80 (8–16, SD 2.59)	10.77 (5–16, SD 2.84)	**8.70** **(4–19, SD 3.34)**	10.42 (4–19, SD 3.18)
D [mm]	57.43 (47–68, SD 6.01)	58.67 (49–66, SD 4.60)	59.07 (48–68, SD 5.36)	58.37 (51–72, SD 4.99)	58.70 (48–72, SD 4.94)
E [mm]	34.91 (25–47, SD 5.58)	35.73 (23–49, SD 5.97)	35.97 (24–53, SD 7.14)	**29.53** **(18–46, SD 6.46)**	33.74 (18–53, SD 7.13)
F [mm]	124.43 (114–138, SD 5.17)	125.90 (119–138, SD 5.09)	125.03 (116–144, SD 6.67)	126.67 (117–139, SD 5.47)	125.87 (116–144, SD 5.75)
G [mm]	132.87 (127–146, SD 5.26)	134.97 (127–151, SD 5.25)	132.97 (120–144, SD 6.67)	135.80 (126–147, SD 6.44)	134.58 (120–151, SD 6.20)
H [mm]	147.35 (141–158, SD 4.84)	149.70142–162, SD 5.40)	146.57 (129–158, SD 7.21)	150.23 (139–164, SD 7.05)	148.83 (129–164, SD 6.73)
FI [mm]	8.87 (4–14, SD 2.32)	8.50 (4–13, SD 2.13)	**7.20** **(3–13, SD 2.47)**	**6.00** **(3–12, SD 2.02)**	**7.23** **(3–13, SD 2.42)**
ICR [mm]	11.35 (7–16, SD 2.44)	10.30 (7–15, SD 1.99)	**8.03** **(4–14, SD 2.70)**	**7.17** **(3–12, SD 2.45)**	**8.50** **(3–15, SD 2.72)**
ICL [mm]	11.83 (8–17, SD 2.61)	10.53 (7–15, SD 2.27)	**8.70** **(4–15, SD 2.88)**	**7.33** **(4–11, SD 2.31)**	**8.86** **(4–15, SD 2.80)**
SW [mm]	7.30 (5–11, SD 1.77)	6.57 (4–9, SD 1.43)	**5.60** **(2–10, SD 1.85)**	**5.47** **(3–11, SD 1.87)**	**5.88** **(2–11, SD 1.78)**
I [mm]	16.48 (12–22, SD 2.31)	16.53 (12–22, SD 2.50)	16.80 (13–25, SD 2.70)	15.80 (11–22, SD 2.81)	16.38 (11–25, SD 2.68)
CFW [mm]	3.83 (1–7, SD 1.70)	3.37 (2–6, SD 1.13)	**2.87** **(1–6, SD 1.41)**	**2.60** **(1–6, SD 1.52)**	**2.94** **(1–6, SD 1.39)**
F/A frontal horn index	2.94 (2.24–3.82, SD 0.40)	**3.27** **(2.80–3.97, SD 0.30)**	**3.33** **(2.5–4.1, SD 0.40)**	**3.47** **(2.66–5, SD 0.52)**	**3.36** **(2.5–5, SD 0.42)**
A/G Evans index	0.32 (0.25–0.41, SD 0.04)	**0.29** **(0.24–0.34, SD 0.03)**	**0.29** **(0.22–0.36, SD 0.04)**	**0.27** **(0.19–0.35, SD 0.04)**	**0.28** **(0.19–0.36, SD 0.03)**
D/A ventricular index	1.36 (1.06–2.00, SD 0.22)	**1.53** **(1.16–1.82, SD 0.17)**	**1.57** **(1.24–1.91, SD 0.19)**	**1.60** **(1.18–2.33, SD 0.28)**	**1.56** **(1.16–2.33, SD 0.22)**
H/E cella media Schiersmann index	4.32 (3.24–5.80, SD 0.66)	4.29 (3.29–6.39, SD 0.69)	4.23 (2.83–6.08, SD 0.83)	**5.32** **(3.26–8.11, SD 1.14)**	4.61 (2.83–8.11, SD 1.03)
A + B Huckman number [mm]	68.74 (52–88, SD 10.01)	**63.57** **(51–77, SD 7.07)**	**60.60** **(46–78, SD 7.98)**	**57.90** **(35–74, SD 9.64)**	**60.69** **(35–78, SD 8.53)**

The values in groups below 100 years of age, reflecting statistically significant differences from the values in the group of 100–106 year olds are given in bold print.

**Table 2:** Correlations between brain atrophy parameters/indices and age in the group of all the patients aged 70–106.

Brain Atrophy Parameter/Index	Correlation Linear Formula	Correlation *r* Coefficient	*p*
A [mm]	24.2400 + 0.1699 * Age	0.31	0.001
B [mm]	6.1862 + 0.1960 * Age	0.45	0.0001
C [mm]	1.7881 + 0.1015 * Age	0.33	0.0001
D [mm]	63.9310 − 0.0629 * Age	−0.12	0.192
E [mm]	18.6610 + 0.1757 * Age	0.26	0.005
FI [mm]	−1.2420 + 0.1010 * Age	0.41	0.0001
ICR [mm]	−4.4330 + 0.1550 * Age	0.54	0.0001
ICL [mm]	−4.3660 + 0.1586 * Age	0.54	0.0001
SW [mm]	−0.0926 + 0.0718 * Age	0.39	0.0001
I [mm]	14.6570 + 0.0200 * Age	0.08	0.410
CFW [mm]	−0.7417 + 0.0443 * Age	0.30	0.001
F/A frontal horn index	4.7410 − 0.0168 * Age	−0.38	0.0001
A/G Evans index	0.1598 + 0.0015 * Age	0.39	0.0001
D/A ventricular index	2.2436 − 0.0083 * Age	−0.36	0.0001
H/E cella media Schiersmann index	7.5399 − 0.0343 * Age	−0.36	0.0001
A + B Huckman number [mm]	30.4270 + 0.3659 * Age	0.40	0.0001
